# Supplementary material for: Tunable DNA Origami Nanosensors for Detection of Multiscale Spatial Ion Concentration Gradients
Source: ACS Sens. Author manuscript; Available in PMC 2026 Jul 28. (PMC13411217; doi:10.1021/acssensors.5c03834)
Supplement: Supporting Information [file NIHMS2194398-supplement-Supporting_Information.pdf]

Supporting Information

Tunable DNA Origami Nanosensors for Detection of Multiscale Spatial Ion Concentration Gradients

Peter E. Beshay[a+], Zachary Osborn-King[b+], Marissa C. Kruse[a+], Jordin T. Marshall[b], Teng Teng[a], Jonathan W. Song[a,c] , Carlos E. Castro[a,c]*, Benjamin A. Walter[b,d]*
[a]Dept. of Mechanical and Aerospace Engineering, The Ohio State University, Columbus, OH 43210, USA
[b]Dept. of Biomedical Engineering, The Ohio State University, Columbus, OH 43210, USA
[c]Comprehensive Cancer Center, The Ohio State University, Columbus, OH 43210, USA
[d]Dept. of Orthopaedics, The Ohio State University, Columbus, OH 43210, USA
*Co-Corresponding Authors: B.A.W. - walter.367@osu.edu; C.C. - castro.39@osu.edu
[+]P.E.B., Z.O.K., and M.K. contributed equally to this work

Supplemental Figures and Tables

Supplemental Table 1: Table of DNA sequences for the modulating and fluctuating linker for all four versions of the device. Helix 0, 3, 6, 9 and 12 are the modulating linkers and are therefore unconstrained when the corresponding unconstrained sequences are included or single stranded when the unconstrained sequences are excluded. Helix 15 is the fluctuating linker containing the donor-acceptor fluorophore pair with the controllable overhang length measured in base pairs.

Name	Sequence	Description
9bp-Cy3	/5Cy3/ATTCTACTATCTGCGAACGAGTAGATTTAGTTTGACCATTAGATACATT	Sequence containing the donor fluorophore for the 9bp overhang on the fluctuating linker
9bp-Cy5	TATATTTTCATTTGGGGCGCGAGCTGAAAAGGTGGCAT/3Cy5Sp/	Sequence containing the acceptor fluorophore for the fluctuating linker for the 9bp version of the device.
10bp-Cy3	/5Cy3/AATTCTACTATCTGCGAACGAGTAGATTTAGTTTGACCATTAGATACATT	Sequence containing the donor fluorophore for the 10bp overhang on the fluctuating linker
10bp-Cy5	TATATTTTCATTTGGGGCGCGAGCTGAAAAGGTGGCA/3Cy5Sp/	Sequence containing the acceptor fluorophore for the fluctuating linker for the 10bp version of the device.
11bp-Cy3	/5Cy3/CAATTCTACTATCTGCGAACGAGTAGATTTAGTTTGACCATTAGATACATT	Sequence containing the donor fluorophore for the 11bp overhang on the fluctuating linker
11bp-Cy5	TATATTTTCATTTGGGGCGCGAGCTGAAAAGGTGGC/3Cy5Sp/	Sequence containing the acceptor fluorophore for the fluctuating linker for the 11bp version of the device.
12bp-Cy3	/5Cy3/TCAATTCTACTATCTGCGAACGAGTAGATTTAGTTTGACCATTAGATACATT	Sequence containing the donor fluorophore for the 12bp overhang on the fluctuating linker
12bp-Cy5	TATATTTTCATTTGGGGCGCGAGCTGAAAAGGTGG/3Cy5Sp/	Sequence containing the acceptor fluorophore for the fluctuating linker for the 12bp version of the device.
Unc. Helix 0	TTTGAGGATTTAGAAGTATTAGACTTTACAAACAATTCGACAACTC<br>TCTAAAATATCTTTAGGAGCACTAACAACTAATAGATTAGAGCCGT	Top and bottom sequence for the unconstrained modulating linker on Helix 0
Unc. Helix 3	AGACGCTGAGAAGAGTCAATAGTGAATTTATCAAAATCATAGGTCT<br>TTAACCTCCGGCTTAGGTTGGGTTATATAACTATATGTAAATGCTG	Top and bottom sequence for the unconstrained modulating linker on Helix 3
Unc. Helix 6	TCGGCTGTCTTTCCTTATCATTCCAAGAACGGGTATTAAACCAAGT	Top and bottom sequence for the unconstrained modulating linker on Helix 6

	CCTGAACAAGAAAAATAATATCCCATCCT AATTTACGAGCATGTAG	
Unc. Helix 9	GTATGGGATTTTGCTAAACAACTTTCAACA GTTTCAGCGGAGTGAG ACTAAAGGAATTGCGAATAATAATTTTTTC ACGTTGAAAATCTCCA	Top and bottom sequence for the unconstrained modulating linker on Helix 9
Unc. Helix 12	CTTAGCCGGAACGAGGCGCAGACGGTCA ATCATAAGGGAACCGAAC ACGGAGATTTGTATCATCGCCTGATAAAT TGTGTCGAAATCCGCGA	Top and bottom sequence for the unconstrained modulating linker on Helix 12

Supplemental Table 2: Table of DNA sequences for all staples used to form the open and closed barrel of the NanoDyn structure when combined with the p8064 scaffold. The bottom barrel is the closed end while the top barrel is the open end.

Sequence	Location
ATGCGTTCTAGCTGATAAACAGGAAATCCTGGAGGCCGCTAT	Cap End
TAATGAGAGATCTACAATCAATCAAAAGAGTAACTATC	Cap End
TTTCAGACAACACCAGTAATGGATCATCAGAGCTGGTCTGGTCAGTCCG	Cap End
CCTAAAGCGTTAAAAGGTTTTGACGTCACTGCTTGTAGAACGTCATTTC	Cap End
AGTGTTGGAACGCGTTAGTATCGCTCAAGAACGCGCCTGTTTATCAACA	Cap End
GAATAGCAATATATTAATTACTGAGAATACAACATGTTCAGCGGGTAAA	Cap End
AAAATCCTCTTCTGTAAGAATACGCCAACTGTCCAGACGACGTCATAAA	Cap End
GATGGTGGTTTGAAATACCCAGTCGGGACGAAAATTTTGGGGGCAA	Cap End
TGGAGCACTAGTGCCACGCCGGAGAGGGTAGGATAATCAATC	Cap End
GCGGTCCTAATGAAACTCACAACCGAGCGTTCTTCGCGTCCGCTGC	Cap End
TCAGAACTGAAAAACAGCAGGTCATTGCCTGGGTAATCAATT	Cap End
CTGATTGGGGCGCCAAGCATAGTGAAATTTTTCACGGTCATAGCGC	Cap End
AATCGCAAGACAAATTCCAGTTTGG	Cap End
TTTTTCACCGAGATAGGGCGATGGTCAGTAAAGATTCAAAAGGAAGCCT	Cap End
AATTTCACTTATAAGGGCGATATCAAACAAGGCCGGAGACAGCCCTGTA	Cap End
TTTAATGGTTCCGAACCATCATCACCTTACCATCAATATGATGTTGTAC	Cap End
TGTCTCACTGATCAGTTGAGGATCCCCGGGTTTAATTGGGCG	Cap End
TCGACCGTGTGATAAGAGGCACCGACAA	Cap End
GCCATGGTCATTTCTGCCAGCACGCCCCCTGTATTTACTCTGCTG	Cap End
TATAAAGCCAACATATGCGTTA	Cap End
GCCAGTACCCGCTTGGGAAGAAAGCCGGACGCCAGAGATTGT	Cap End
AAGGTAAGTGTGTTCAGCAAACGCAACCAGCTTACACCGATA	Cap End
CCTCAAAGAGAATACGGCATCCCGCCGCGCGCGTAATTAAAGGGCG	Cap End
GGGTAGCTGTTTCCTGTAAGTGTACCAGTAAAAGAATATCAC	Cap End
TCACTGTTGCCCTGCGGCCAATCCGCCGGGCGCGGT	Cap End
AATTAATGCACAGTAGGGCTTAATTAGAAAAAGCCTGTAGAAAAC	Cap End
GGTTTCTCATTGCAGGCGCTTTTATCAGTCGCTGAAACGAGGCATCTTT	Cap End
TCGACAATAACGCCATATTTAACAAAACACCGGAATCATTTAGTT	Cap End
CATCCCTGGTGTCCAGCATCAGAATTTCACGCATAAGAGGCTAAAAGAA	Cap End
CTGAGTAATTCATGTAATTTAGGCAATAAGGCGTTAAAACCTAAA	Cap End
AGCCGCTGCGACGAGCACGGGAGCCGCTATTGTCGGATTCTCCGTCGTT	Cap End
AGTGCTCAATAATATTAAGTAGAAGATTAAGAGCCAGC	Cap End
AACGCCGGTGCGTGCCTTCGAATTCGTAATCTAATGAGGCAG	Cap End
GGACGCGCCTCGGGCCGTGTTATC	Cap End
TCCAAAAGGAGCCTTAAAGGCCGCT	Cap End
GTTGCACAGGCGGCCATCCCATCGTTAATAAAGTATTTTCGA	Cap End
AGTCCGTAAAATTAAACGGGTACAATCGGCGGACATAATGCT	Cap End
ACGGTACATCAAACGTAATAACCTCACCGGAGCCTCTTTAAA	Cap End

GCACGCCAGCCCAGTCCGTGGAGCCGCCACGCTGGCGACGTT	Cap End
GTCCTTAAATGATAGACGCCAGTGCCAAGCTGCAAGGCGAACTCACTGT	Cap End
CCTCAGCAGCGATACCAAGCGCGAA	Cap End
GCATCGGGGCTTGCAGGGAGTTTAATTGTATCGGTTCGCACTTGGT	Cap End
CGGCTACACCGATATATTCGGCTTGCTTTCGAGGTGCGGGGTTTGC	Cap End
CTAAAGAAACAACCATCGCCCTTAAACAGCTTGATGGCTGGATACA	Cap End
AGTTTCCAAAAGCCGCGCCGACAATGACCTTTTTCCCAACCT	Cap End
GACCCCCGGCACCGCTTCTGGATCTGCCAGTTTGAATCATACAACG	Cap End
TACACTAAACCAGGCAAAGCGATGGGCGCATCGTAATTAGCATGCG	Cap End
AAAACGACCATTCAGGCTGCGGGATAGGTCACGTTAGCCTCAATTA	Cap End
GTTGTAAACGCCAGACATCACCGTTGTATGACCTGAAAACATGCAACAG	Cap End
CACTCCAGCCAACGACAGTATC	Cap End
ATCAACGGCGGATTGACATCAAATATTTAAA	Cap End
CCCACGCCAGGGAACGGCAGCGCCATGTTTAAGTTGGGCGTG	Cap End
TTAATGTGCTTTCAGAGCGGAATTTGTGAGATTCTGCTCCAG	Cap End
TTTCATCCAGCTCATAGCATGAGGCTATAGGTGAGGCGGTCAAGTC	Cap End
TGTTTGGGTAAACGACGTTTCTCCGTGGTGAAACA	Cap End
TCTGGCCGGAACGCGATGAACAGAGTCTCACCAGCAGAAGATTAGC	Cap End
AGTAGCATTAACATCCAATAAGGGGACGGCTTTCCAGCGATTAAAGACA	Cap End
TTGTAAAGGGAACAGGTGCGGAAAAATACGTAATGCCACTACTGGGAAG	Cap End
AATCCCCAAAAATTAATGCTGAGAGCCAGCATCGAGGTACGG	Cap End
CAAGTGTAGGTTGGCAAATCAACAGACTCCAACGTCAAAGGGTTG	Cap End
TTATTTCAGGCAAGGCAAAGAACCGTGCTGCCGGAAAACACTGTAGCAA	Cap End
GGAGGTGAGACCTCAATCAATATCAAAACCGTCTATCAATCAAAA	Cap End
ATACTTTAAATTAAGCAATAAGGTGTAGCCATTCGAAGAGGCTTGAGGA	Cap End
TGATCAAATCGCTGAACCTCAAATGGCCCACTACGTGAAATCGGC	Cap End
CAAAAACGAGCATAAAGCTAACGTAATGCAACTGTGAAGGCAATGAGGA	Cap End
ATAAGCGATTCAACAAAAATCTAAAGCACCCAAATCAAGTTTCCTGTTT	Cap End
ATGCAATGCCTGAGTAATGGATAAAAATTTTTAGAA	Cap End
TTTCACCGCCTGCAACAAATCGGAACCCTATGCAGCAA	Cap End
GGAAAGCGAAACCACACAGATGCCACCGTCGGTGGTGCCTTT	Cap End
CTACGATTTATTTTTATAGAAAAGATTTTGTTAAAATTACAA	Cap End
GGCAATGTGAGCTATCGGCCAACGCGCGGAGAGTTGGCTATTGTAT	Cap End
ATTTATTTTTCCGAGTATATGTACAATTTTTGTTAAATAACA	Cap End
ACCTACAGACATTCGAGCCGGAGGGTGG	Cap End
GGTAAAAATCGATGAAGGGTAAAGCGGCAGCGGGTTACTGAGCCT	Cap End
TTGCGTAACCAGGAGCGCGTTGCGCGTGCCAGCTGCATACGC	Cap End
CTTATGTGTAGCGGTCACAGCGGTCGCAAGAATGCCAATTAA	Cap End
GTAATATCATTTGCAACGCGGTCCGTTTGCGTGGTCGATCCACCGG	Cap End
AACCGTCTGACACACGAAAGCCTGGCGGTTTGCGTATTCCCT	Cap End
CAGAGAAGTGGAGCTTGGCCGTAATTTGCCCCAGCAGGAACC	Cap End
AGAGAGGCCAGAAAAGGGAGCCCCGGGCGCT	Cap End
GGCCTCTAAGGGGGAATGTGAGCGAGTACGCATTACCCGGTTCTAT	Cap End

AGTATCAGAGCGTATAACGGTTGTATGCTGATTGCCGTCAGC	Cap End
CCACGTGGCAAATGCGCCCGCCTGGCCCTGAGGGAGAGGGGT	Cap End
GCAGTAAAACTTTTTTAACCAATATTCC	Cap End
AACTTGCCTGCCGCCAGGATCAAATCGTCGCTGGCAGCTGCC	Cap End
TGAATAATTTGCACGTAAAACAAGCGCATTAG	Open End
TCACATCAATCAGTTCAGAAAACGCGAGAGGCTTTTGCAACCCTC	Open End
ACATAAATCAGAGAAATAGCAGAAACGCAACATATAAAAGAAATATGGT	Open End
GCAGCCTATTGAGTTAAGCACGATTTTTTGTTTACCCAATCCTAAGACT	Open End
CAAAAATTGAATATGATTATCCCATAAATCAAAAACGGATGGCTAAACC	Open End
AATTTTTACACGGAATTTTTACCCTGACTATTTTGATA	Open End
TTACCTTGTGAGTGTAAACAGAAGAACGATATAGAAGGCTTAATATTGA	Open End
CATTTAATGTAAATCCTAATTGAACCTCGCCCAATAGCAAGCTAAAGGT	Open End
TCAAGAAAATTTTCCTAACGAGTTTTGACATCGTAGGAATCAACCGACT	Open End
GCAAAAGAAGAAAAACATAGCGATA	Open End
GCTAATAAACAGGGAGAAATATTGGATTATCCCCCTCAAATGGTTTTGC	Open End
TTGCTTCCAATTTCAACGGATCAAAGAAAGAAGCAAAGCGGAAGAGTAC	Open End
ATTAATTAACAAAATGAATACTAACATTAAAAAGATTAAGAGACTCCAA	Open End
ATCCTTGTGATGAAGCGCAGATATTAATGAAAGACTTCAAATATCG	Open End
ACAATGAGATAACCCACAAGATTACAGAATTT	Open End
AATAAGAACCCAAAAAATACATACATAAGACAAAA	Open End
AATTTTATCCTATTAGTTGCTA	Open End
AGTTACCAGAAGGAAACCGAGATAGCTATCTTACCATTGAGC	Open End
CCTTACGGTATTCTCCATATTATTTATCATAAATCAATATATTTTT	Open End
TTAGCAACGATTGAGGGAGGGCAGACTGTAGCGCGCATTTCGTCACCA	Open End
TCGAGAACAAGCAAGCCGTTTATTAGAGCCAGCAAAATC	Open End
TCAATAGAAAATTCACGCAAAGACACCA	Open End
TTACCAGCCTTATTAGCGTTTCTCCCTCCGCCGCCACAAATAGTAAGCG	Open End
AAAAGGTGGCAATAATAACGGAATGCAAGAA	Open End
GGGCGACCGGCATTTTCGGTCCAGAACCCACCAGACCAGAAT	Open End
CGGAAATAATCAAGTTTGCCTTACAACGAGGAACCTTGCTCAACCGGAT	Open End
CATAAATCAGCGAGGCGTTTTAGCTGCCAGTTACAAAAAATAACC	Open End
GAATTATCACCGTAATCAGTACAGACAGTTCAGGGAGTGCCGTCAAGAG	Open End
GTCTTACCGCCCGACTTGCGGGAGGCGTCTTTCCAGAGCGTCGCT	Open End
TGAGCCACAATGAAACCATCGGTAACGACTCAGAGATAAGTAGCATAGG	Open End
GGATTATTTTAGCCTTAAATCAAGGAATCTTACCAACGCCTTAGA	Open End
CATATTCAACACGTAGAAGAACTGGCATGATAAATAAGAACCAAT	Open End
CCATTAGCAAGGCCGGAAGTCTTTCGCCACCCTGTATCATGAACGG	Open End
TACAAACTTAGCGTAAGGTAATCTTACGCAGTATG	Open End
CTGAGCCAGGATTATAAGAGGCTGAGACCCTATTTCGGAACCATAAAAC	Open End
CCCTCAGAACCCAGACGTTAGTAAA	Open End
CTTGATATTCACAAAGCATTGACAGGAGACCACCG	Open End
ATTAAAGACCACCACCAGAGCAGAGCCGCCACCCTATAGCCCCGCC	Open End
GGAAACTCAAGAGAACCCTCAGAGCCGCGCCACCCTCAGAGCTTTT	Open End

GCGGATAATAGCAAGCCCAATCCTGTAGCATTCCAGCGACAGTATT	Open End
GGTTGATCCACCACCCTCATTCCCTCATAGTTAGCATAGCAGCACC	Open End
GAATAGGTCAGAACCGCCACCTCTAAAGTTTTGTCACGTCACTTTG	Open End
TCATACAGCCTTGAGTAACAGTTCATCAGTTGAGATTTA	Open End
AATTTACATAAACAGTTAATGACATTATTACAGGTCTATCATAAAA	Open End
ATACATGAAAGTATGGATTAGCGGGGTTCATGTACCGTAACA	Open End
TAATCTTCGTAACAAAGCTGCTCATTATACCAGTCGCTCAACTGCT	Open End
CTGGCTGGTGAATAAGGCTTGTATGCGATTTTAAGCAACTAAGGAT	Open End
TGTACAGGAGAAACACCAGAATTTAATCATTGTGAAAGTTTCGAAG	Open End
TGAAAGAGGACAGACCGTACTCAGG	Open End
CTGAATCATTACCCTGGGAAGAAAAATCATTGCTGATTTTTGTCAG	Open End
GAACTAAAGACGACGATAATAGAGCTTATACGTTATATTATT	Open End
TTAATTTCAACCGAGTAGTAAA	Open End
GGAATTACGAGGCAGGTAATATCATTGAATACTTC	Open End
GTTTACCCGGAACACCCCCTGTCGCGCAGTCTCTG	Open End
CAGAGGGTAGTAAGAGCAACAAGAAAGATGCCCGTCGTTCCAAATCCTC	Open End
GAACTTTAAAATAATCCTGATTGTAAGAAATTGCGTAGGAGAATA	Open End
AAAATAGAGAATGAAGATGATGGCAATTGGTTTAACGTCAGAGAAAATA	Open End
AGAGGTCAATATAATGCTGTAAGGACGTAAATCAAGACAAGAGTACCAG	Open End
CCTTATAGTCACCACCAGAAGGAGTCGGGAGAAACAATATTTGAA	Open End
CTTTAATATGTTTTAAATATGAACTGGCTCATTCAACCTTCATCGAGAG	Open End
TAGTTGCATCATCATTTTGCGGAATCGCCTGATTGCTTTTAATTA	Open End
CAGGTCAAGTACGGTGTCTGGATTACCTCCCTGACACCAGGCTAGCCC	Open End
CAAGAAGCCCTTTAAAAGTTTGAGCAAGTTACAAAATCACAAACA	Open End
TCAAAGCGAACCAGACCGATTCCATATAACAGTTGATTC	Open End
GTCATCATCATATTCCTACAGTAACAGTACCGGAAACAGTAACGT	Open End

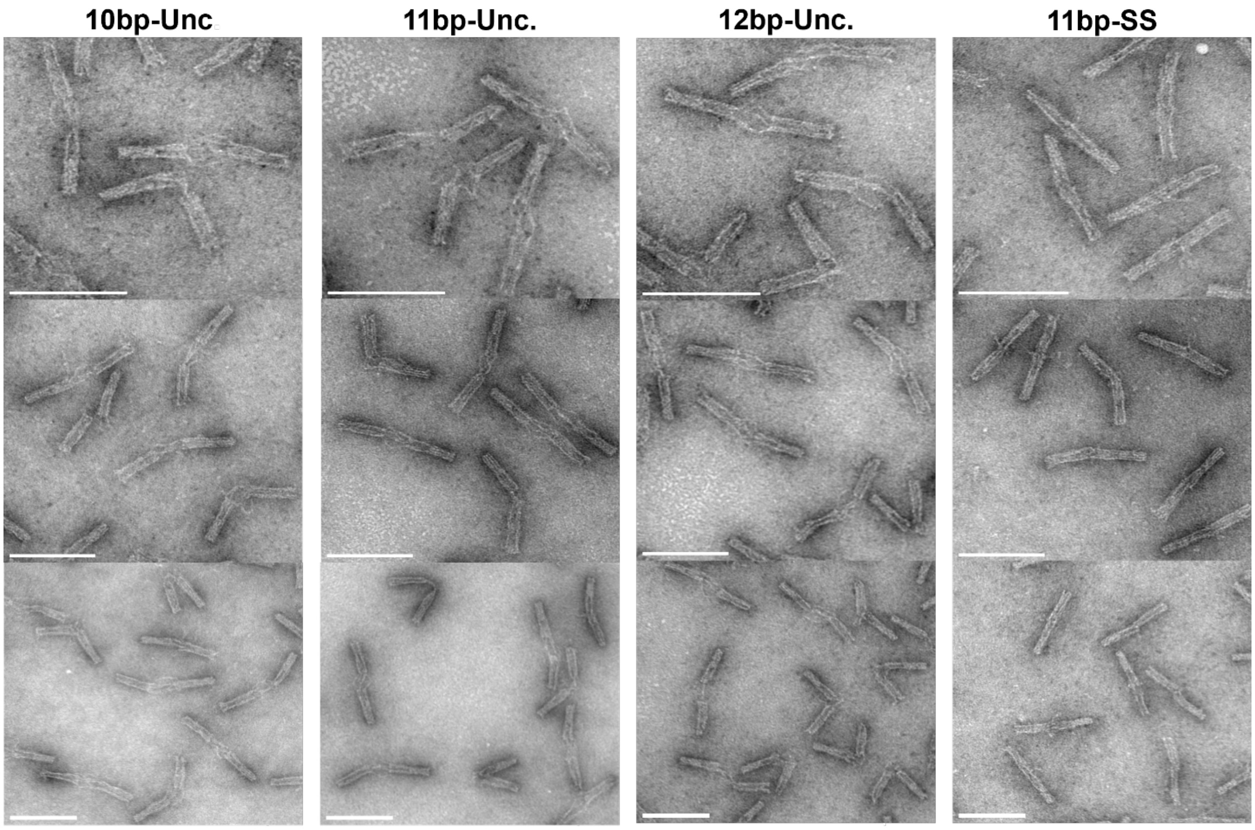

Supplemental Figure 1: Images of the different versions of the DNA origami structures tested at varying magnifications to visualize the stable state of the structure in 300mM NaCl + 10mM MgCl2. The magnification that each image was taken at starting from top to bottom is: 150kx, 100kx, and 60kx. As explained in the text and shown in the image, as more base pairs are added going from left to right, the structures tend to favor the closed stable state over the open stable state. Each scale bar represents 100 nm.

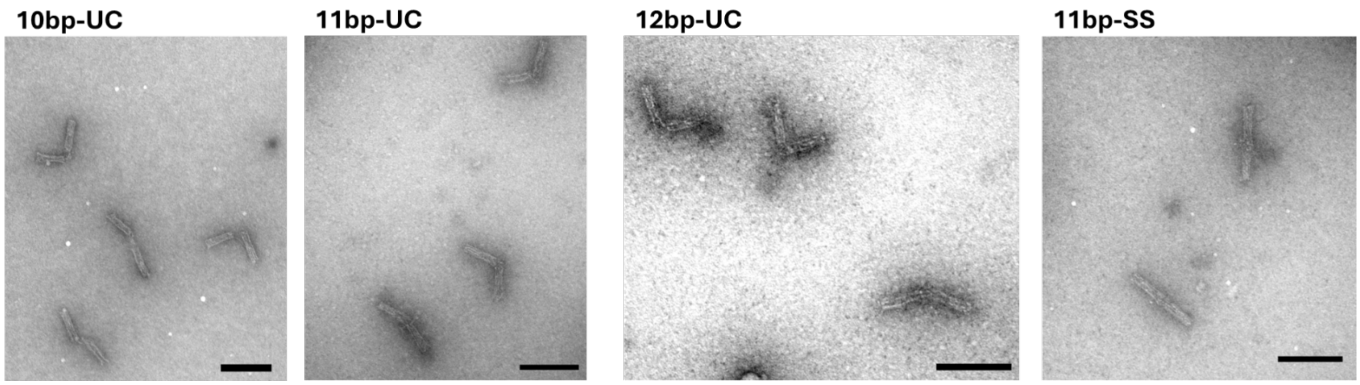

Supplemental Figure 2: TEM Images of the different versions of the DNA origami structures imaged at 300 mM NaCl and 1 mM $MgCl_2$. Each scale bar represents 100 nm.

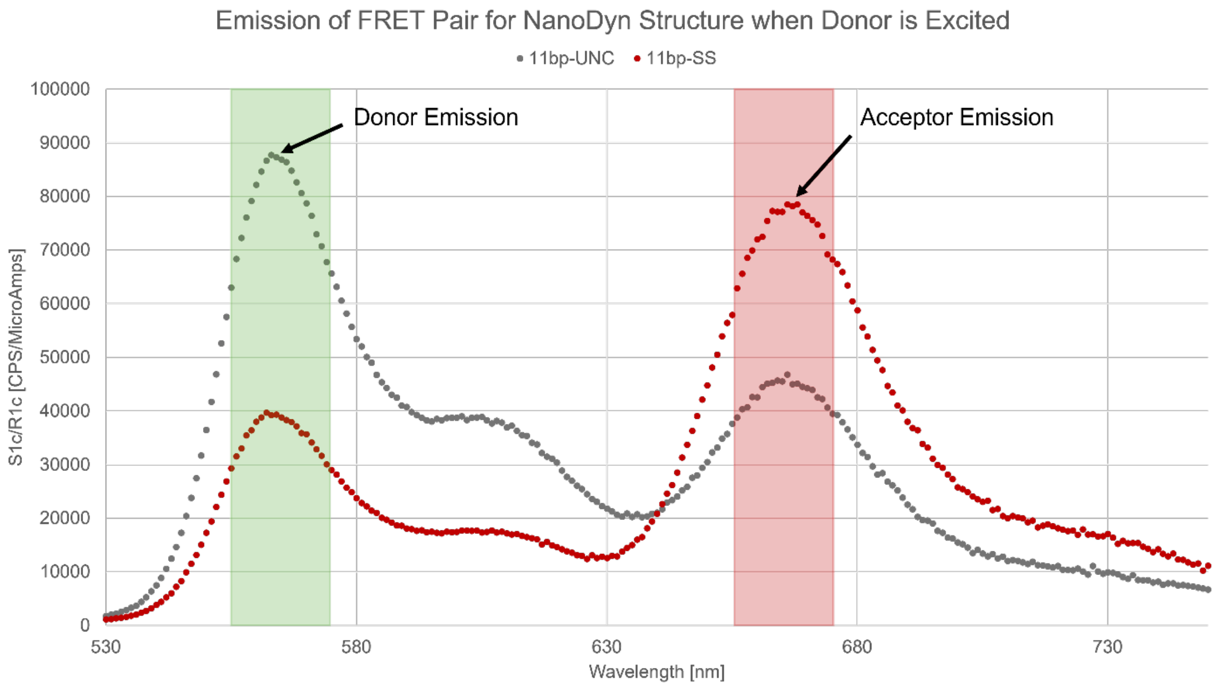

Supplemental Figure 3: Graph of the FRET pair emission on the fluorometer when the donor is excited directly at 510 nm. The graph shows the difference in emission peaks for the two variations of the device: 11bp-UNC and 11bp-SS. As the device shifts from the open configuration to the closed configuration, the acceptor emission peak overcomes the donor emission peak. Therefore, the 11bp-SS device favors the closed state more than the 11bp-UNC version which is confirmed by our TEM images.

Supplemental Table 3: Sensor Design Parameters Effect Sensitivity to Salt Concentration

Design Version	Initial Sensitivity (dFRET/dC @ 100mM) [1x10^{-3}/mM]	Saturation Constant (C_{Sat}) [mM]	Effective Range (Max: 100+2*C_{sat}) [mM]	Average R^2 of Fit	Average RMSE of Fit [1*10^-3]
10bp-UNC	0.19 ± 0.03	788± 133	100 – 1675	0.99 ± 0.003	3.42 ± 0.93
11bp-UNC	1.23 ± 0.24	133 ± 9.6	100 – 365	0.98 ± 0.014	7.69 ± 1.44
12bp-UNC	1.45 ± 0.12	174 ± 16	100 – 448	0.99 ± 0.005	6.99 ± 2.51
11bp-SS	3.13 ± 0.50	69 ± 6.6	100 – 238	0.99 ± 0.005	8.03 ± 0.71

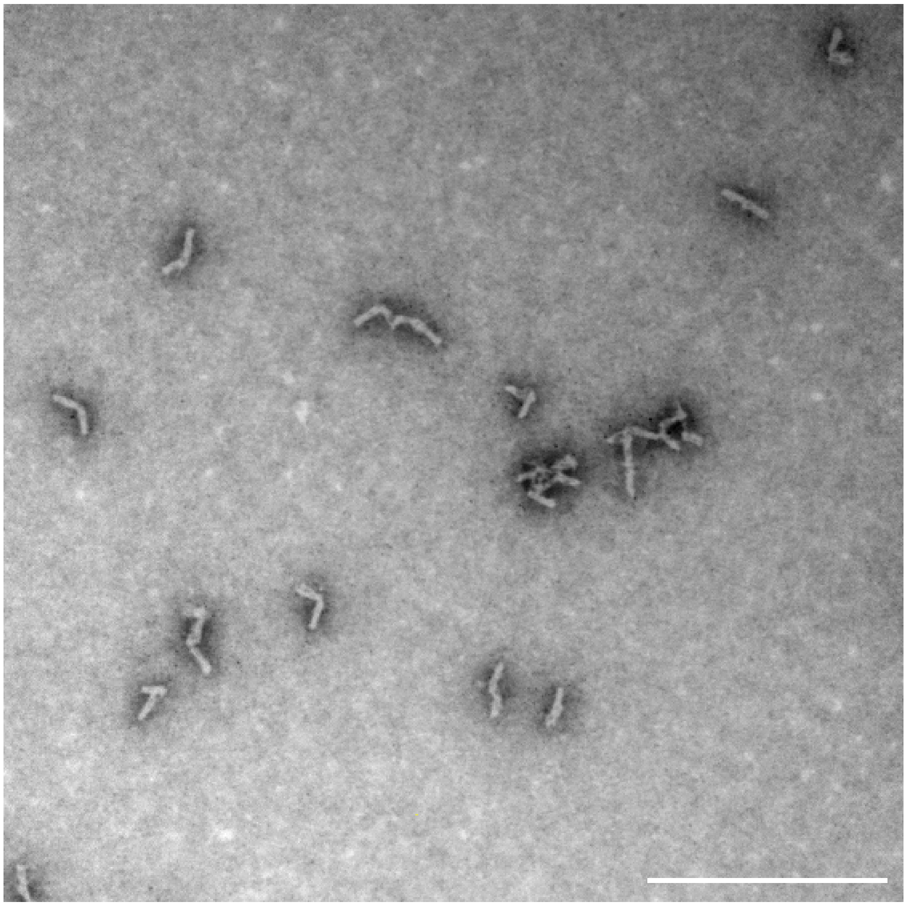

Supplemental Figure 4: Image of 11bp-UNC DNA origami structures from the outlet channel after being flown through the microfluidic device with inlet conditions of 450-150-150 mM NaCl + 1mM $MgCl_2$. This image confirms that the devices remain intact throughout the duration of the experiment. The scale bar is 500 nm.

Supplemental Table 4: Sensor Design Parameters Effect Spatial Sensitivity to Salt Concentration

Concentration Profile (mM)	Concentration Change (mM)	Max Slope (FRET Eff*10^{-3}/μm)		Distance to Equilibrium (μm)	
		**11 UNC**	**11 SS**	**11 UNC**	**11 SS**
150 to 450mM	Δ300mM	11.39 ± 4.09	3.36 ± 1.0	6.56 ± 1.86	12.66 ± 3.06
150 to 350mM	Δ200mM	6.90 ± 2.53	4.36 ± 1.50	7.69 ± 2.72	9.15 ± 3.29
150 to 200mM	Δ50mM	2.00 ± 0.51	2.57 ± 0.73	9.32 ± 1.84	9.93 ± 2.88
150 to 175mM	Δ25mM	1.23 ± 0.39	1.56 ± 0.33	7.67± 2.53	9.51 ± 2.75
